# Association between brain amyloid deposition and longitudinal changes of white matter hyperintensities

**DOI:** 10.1186/s13195-024-01417-8

**Published:** 2024-03-07

**Authors:** Woo-Jin Cha, Dahyun Yi, Hyejin Ahn, Min Soo Byun, Yoon Young Chang, Jung-Min Choi, Kyungtae Kim, Hyeji Choi, Gijung Jung, Koung Mi Kang, Chul-Ho Sohn, Yun-Sang Lee, Yu Kyeong Kim, Dong Young Lee

**Affiliations:** 1 Department of Neuropsychiatry, Seoul National University Hospital, Seoul, Republic of Korea; 2 Institute of Human Behavioral Medicine, Medical Research Center, Seoul National University, Seoul, Republic of Korea; 3 Interdisciplinary program of cognitive science, Seoul National University College of Humanities, Seoul, Republic of Korea; 4 Department of Psychiatry, Seoul National University College of Medicine, Seoul, Republic of Korea; 5 Department of Psychiatry, Inje University Sanggye Paik Hospital, Seoul, Republic of Korea; 6 Department of Radiology, Seoul National University Hospital, Seoul, Republic of Korea; 7 Department of Nuclear Medicine, Seoul National University College of Medicine, Seoul, Republic of Korea; 8 Department of Nuclear Medicine, Seoul Metropolitan Government-Seoul National University Boramae Medical Center, Seoul, Republic of Korea

**Keywords:** Alzheimer’s disease, Beta-amyloid, Tau, White matter hyperintensity, Sex difference

## Abstract

**Background:**

Growing evidence suggests that not only cerebrovascular disease but also Alzheimer’s disease (AD) pathological process itself cause cerebral white matter degeneration, resulting in white matter hyperintensities (WMHs). Some preclinical evidence also indicates that white matter degeneration may precede or affect the development of AD pathology. This study aimed to clarify the direction of influence between in vivo AD pathologies, particularly beta-amyloid (Aβ) and tau deposition, and WMHs through longitudinal approach.

**Methods:**

Total 282 older adults including cognitively normal and cognitively impaired individuals were recruited from the Korean Brain Aging Study for the Early Diagnosis and Prediction of Alzheimer’s Disease (KBASE) cohort. The participants underwent comprehensive clinical and neuropsychological assessment, [^11^C] Pittsburgh Compound B PET for measuring Aβ deposition, [^18^F] AV-1451 PET for measuring tau deposition, and MRI scans with fluid-attenuated inversion recovery image for measuring WMH volume. The relationships between Aβ or tau deposition and WMH volume were examined using multiple linear regression analysis. In this analysis, baseline Aβ or tau were used as independent variables, and change of WMH volume over 2 years was used as dependent variable to examine the effect of AD pathology on increase of WMH volume. Additionally, we set baseline WMH volume as independent variable and longitudinal change of Aβ or tau deposition for 2 years as dependent variables to investigate whether WMH volume could precede AD pathologies.

**Results:**

Baseline Aβ deposition, but not tau deposition, had significant positive association with longitudinal change of WMH volume over 2 years. Baseline WMH volume was not related with any of longitudinal change of Aβ or tau deposition for 2 years. We also found a significant interaction effect between baseline Aβ deposition and sex on longitudinal change of WMH volume. Subsequent subgroup analyses showed that high baseline Aβ deposition was associated with increase of WMH volume over 2 years in female, but not in male.

**Conclusions:**

Our findings suggest that Aβ deposition accelerates cerebral WMHs, particularly in female, whereas white matter degeneration appears not influence on longitudinal Aβ increase. The results also did not support any direction of influence between tau deposition and WMHs.

**Supplementary Information:**

The online version contains supplementary material available at 10.1186/s13195-024-01417-8.

## Introduction

Alzheimer’s disease (AD) and cerebrovascular disease (CVD) commonly co-occur [1] and have additive effects on cognitive decline or onset of dementia [2, 3]. White matter hyperintensities (WMHs) on T2-weighted magnetic resonance image (MRI) are frequently found in AD dementia as well as CVD. Since WMHs have been related to demyelination and axonal loss caused by chronic ischemia due to cerebral small vessel disease, WMHs in individuals with AD dementia are commonly considered as a marker of comorbid CVD [4].

However, growing evidence suggests that not only CVD but also AD pathological process itself cause cerebral white matter degeneration, resulting in WMHs. Several postmortem studies demonstrated that both beta-amyloid protein (Aβ) deposition and intraneuronal aggregation of phosphorylated tau protein (p-tau) were associated with white matter degeneration via disorganizing myelin architecture [5–7]. A couple of preclinical studies also showed that AD transgenic mice exhibited oligodendrocyte dysfunction and axonal degeneration, suggesting that white matter degeneration may be an important pathophysiological feature of AD [8, 9].

Meanwhile, some preclinical evidence indicated that white matter degeneration may precede or affect the development of AD pathology. An experimental study reported that myelin basic proteins regulated Aβ deposition by binding Aβ and inhibiting formation Aβ fibril and their decline related to the accumulation of Aβ [10]. Other preclinical studies also showed that ischemic axonal injury was associated with increase of tau phosphorylation and neurofibrillary tangle [11, 12].

The relationships between in vivo AD biomarkers and WMHs on MRI were also demonstrated in living human by several cross-sectional studies. Lower cerebrospinal fluid (CSF) Aβ_1−42_, but not with CSF total tau (t-tau) or p-tau, was associated with higher WMH volume [13, 14]. Similarly, higher Aβ, but not tau, deposition on positron emission tomography (PET) was associated with greater WMH burden [15, 16]. Unlike CSF tau or tau on PET, increased plasma tau concentrations were associated with higher WMH volume [17].

However, limited information is available regarding the direction of causal relationship between AD pathology and WMHs in living human brain. Only a couple of longitudinal studies have investigated a causal relationship between WMH and Aβ using CSF or imaging data, with mixed results in both directions [18–20]. Moreover, no longitudinal study investigated the effect of baseline tau deposition on WMH change or the effect of baseline WMHs on tau deposition change yet.

Therefore, this study aimed to investigate the direction of relationship between in vivo AD pathologies, particularly Aβ and tau deposition, and WMHs through a longitudinal approach in older adults with diverse cognitive spectrum including normal cognition, mild cognitive impairment (MCI) and AD dementia.

## Methods

### Participants

This study is a part of the Korean Brain Aging Study for Early Diagnosis and Prediction of Alzheimer’s Disease (KBASE), which is an ongoing prospective, longitudinal cohort study that began in 2014 [21]. Total 282 older adults aging from 55 to 90 years including cognitively normal (CN), MCI and AD dementia individuals were recruited from the KBASE cohort. CN individuals were defined as individuals without MCI or dementia and with clinical dementia rating (CDR) global score of 0. MCI was defined as having CDR score of 0.5 and meeting core clinical criteria for diagnosis of MCI on the National Institute on Aging and Alzheimer’s Association (NIA-AA) guideline [22]. AD dementia was defined as meeting the criteria for dementia on the Diagnostic and Statistical Manual of Mental Disorders 4th edition (DSM-IV-TR) [23] and the NIA-AA criteria for probable AD [24]. All participants with AD dementia had CDR score of 0.5 or 1. The exclusion criteria were the following: (1) presence of a major psychiatric illness; (2) significant neurological or medical condition or comorbidity that could affect mental functioning; (3) contraindications for an MRI scan (e.g., pacemaker or claustrophobia); (4) illiteracy; (5) presence of significant visual/hearing difficulties and/or severe communication or behavioral problems that would make clinical examinations or brain scans difficult; (6) pregnancy or lactation; and, (7) use of an investigational drug. Additional comprehensive participant details were previously provided [21].

### Clinical and neuropsychological assessments

All participants were given standardized clinical assessments by trained board-certificated psychiatrists based on the KBASE clinical assessment protocol which incorporated the Korean version of the Consortium to Establish a Registry for Alzheimer’s Disease Assessment (CERAD-K) clinical assessment [25]. Vascular risk factor scores (VRS) were calculated as the total number of vascular risk factors including hypertension, diabetes mellitus, coronary heart disease, hyperlipidemia, cerebrovascular accident and transient ischemic attacks [26]. All participants were also administered a comprehensive neuropsychological assessment battery by clinical neuropsychologists or trained psychometrists in accordance with a standardized protocol which incorporated the CERAD-K neuropsychological battery [27]. Details on information of clinical and neuropsychological assessments were described previously [21].

### Apolipoprotein E ε4 genotyping and coding

Genomic DNA was extracted from whole blood samples and apolipoprotein E genotyping was performed as described previously [28]. Apolipoprotein ε4 (APOE ε4) positivity was coded as positive if there was at least one ε4 allele and coded as negative if there was no ε4 allele.

### Measurement of cerebral Aβ deposition

**Fig. 1:**
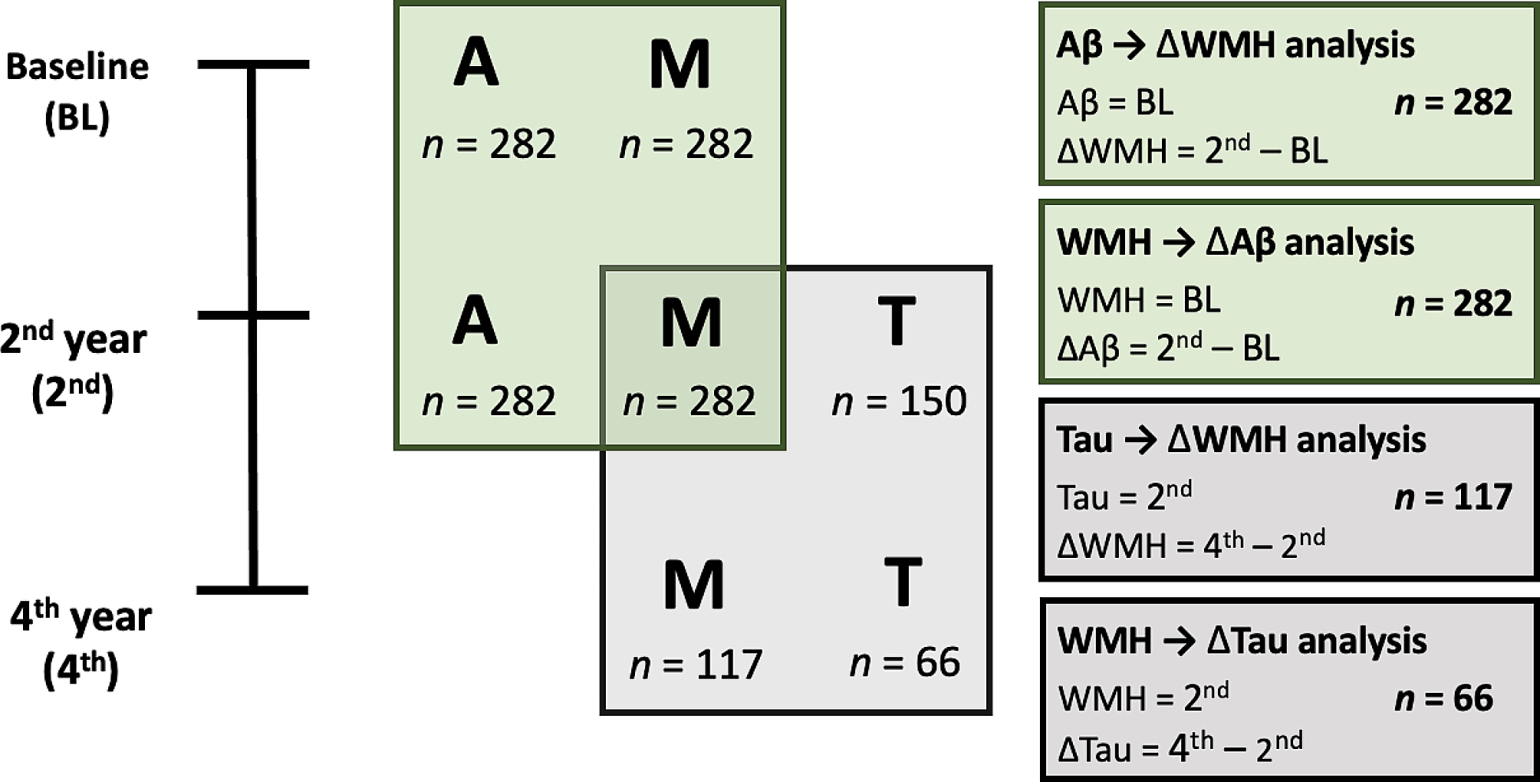
Datasets for analysis. Dataset for analysis of association between Aβ or Tau deposition and WMH volume. Aβ = Beta-amyloid, WMH = White matter hyperintensity. A = Aβ-PET, M = MRI scan, T = Tau-PET

All participants underwent simultaneous three-dimensional (3D) [^11^C] Pittsburg compound B (PiB)-PET and 3D T1-weighted MRI using the 3.0T Biograph mMR (PET-MR) scanner (Siemens, Washington DC, USA) at both the initial assessment and 2-year follow-up (Fig. 1). The image processing was conducted utilizing SPM8. An automatic anatomic labeling algorithm and a region combining method were applied to identify specific regions of interests (ROIs) [29, 30]. To quantify the extent of cerebral Aβ deposition, the uptake value of [^11^C] PiB was extracted from ROIs encompassing the frontal, lateral parietal, posterior cingulate-precuneus, and lateral temporal regions. The calculation of the voxel-weighted mean standardized uptake value ratio (SUVR) for these ROIs was achieved by dividing the mean [^11^C] PiB uptake value of these ROIs by the corresponding mean uptake values of cerebrum white matter, cerebellar white matter, pons and inferior cerebellar gray matter regions [31–33]. More detailed information about the methodology used for measuring cerebral Aβ deposition can be found in a prior publication [21].

### Measurement of cerebral tau deposition

A subgroup of participants (*n* = 150) underwent [^18^F] AV1451-PET scans using Biograph Truepoint 40 PET/CT scanner (Siemens, Washington DC, USA). While all the other neuroimaging scans were performed at baseline and then at 2-year follow-up, [^18^F] AV-1451 PET imaging was first administered at 2-year follow-up time and then repeated 2 years (i.e., 4-year follow-up time) (Fig. 1). Images were coregistered and resliced into T1-weighted MRI for further processing. The computation of the weighted mean SUVR was carried out in native space, incorporating partial volume correction, and normalized by the mean hemispheric cerebrum white matter uptake. This calculation was employed to compute composite ROIs that combined a set of AD-signature regions including entorhinal, amygdala, parahippocampal, fusiform, inferior temporal, and middle temporal ROIs [34]. Further elaboration on the methodology utilized for quantifying cerebral tau deposition can be found in a previous publication [35].

### Measurement of WMHs

Each participant underwent simultaneous 3D fluid-attenuated inversion-recovery (FLAIR) images using the 3.0T Biograph mMR (PET-MR) scanner (Siemens, Washington DC, USA). FLAIR images were obtained at baseline, 2-year follow-up, and 4-year follow-up (Fig. 1). FLAIR images were acquired in sagittal plane, with the following parameter settings: TR, 5000ms; TE, 173ms; echo spacing, 3.46ms; FOV, 250 mm; matrix size, 256 × 256; slice thickness, 1.0 mm. To calculate the volume of WMH on FLAIR images, a previously validated automated approach was employed [36]. The procedure can be briefly summarized as follows: spatial coregistration of T1 and FLAIR images; fusion of T1 and FLAIR images; segmentation of T1; attainment of transformation parameters; deformation and obtainment of the white matter mask; obtainment of FLAIR within the white matter mask; intensity normalization of the masked FLAIR; nomination of candidate WMH with a designated threshold; creation of a junction map; and elimination of the junction. Two modifications were made to adapt for the current data. First, a threshold value of 70, more suitable for our specific data, was applied instead of the original reference value of 65. Second, given the exclusion of participants with acute cerebral infarcts, diffusion-weighted imaging was not integrated into the procedure. WMH candidate images were utilized to extract WMH volumes based on lobar ROIs in the native space for each participant. WMH volumes were log-transformed to reduce skewness (Supplementary Figs. 1 and 2).

### Statistical analysis

To investigate the relationship of AD pathology on WMH volume change over 2 years, we tested multiple linear regression model with brain Aβ or tau deposition on initial PET scan as an independent variable and longitudinal change of WMH volume over next 2 years as a dependent variable controlling age, sex, APOE ε4 positivity, VRS, CDR-Sum of Boxes (SOB) score, and baseline WMH volume as covariates. We also analyzed the regression model with baseline WMH volume as an independent variable and longitudinal change of Aβ or tau deposition for 2 years as a dependent variable controlling age, sex, APOE ε4 positivity, VRS, CDR-SOB, and baseline Aβ or tau deposition as covariates. The longitudinal change of Aβ, tau, or WMH volume was calculated by subtracting baseline value from the value at follow-up, which was represented as delta (∆) (Fig. 1). Additionally, we performed exploratory analyses including interaction term as an additional independent variable in the multiple regression model to investigate the moderating effects of age, sex, APOE ε4 positivity, VRS, CDR-SOB, and baseline value of neuroimaging marker on the relationships which showed statistical significance by any analysis described above. When a significant interaction effect was found, subsequent subgroup analyses were performed. All analyses were performed using R Statistical Software (v4.1.2; R Core Team 2021).

## Results

### Participant characteristics

Table 1 shows the characteristics of the participants. To examine the relationship between baseline Aβ deposition and change of WMH volume over 2 years and vice versa, overall 282 participants were included in the analyses (Fig. 1). For the association between initial tau deposition and change of WMH volume over next 2 years, 117 participants were included in the analysis (Fig. 1). Among them, 66 participants (56.4%), who received the second Tau-PET images at 4-year follow-up, were included in the analysis to investigate the relationship between baseline WMH volume and change of tau deposition over 2 years (Fig. 1).

**Table 1:** Participant characteristics

		Longitudinal analysis			
		Aβ - ΔWMH (*n* = 282)	WMH - ΔAβ (*n* = 282)	Tau - ΔWMH (*n* = 117)	WMH - ΔTau (*n* = 66)
No. (% of sample)					
	Female, *n* (%)	161 (57.09)	161 (57.09)	75 (64.10)	43 (65.15)
	APOE ε4 carrier, *n* (%)	75 (26.60)	75 (26.60)	38 (32.48)	20 (30.30)
	Diagnosis (CN / MCI / AD)	169 / 80 / 33	169 / 80 / 33	53 / 32 / 32	37 / 15 / 14
Mean (standard deviation)					
	Age, y	70.9 (7.90)	70.9 (7.90)	73.7 (7.88)	74.0 (6.97)
	Education, y	11.1 (4.99)	11.1 (4.99)	10.9 (4.91)	10.6 (4.95)
	MMSE score	24.6 (4.21)	24.6 (4.21)	23.4 (5.64)	24.1 (5.02)
	Vascular risk score	2.19 (1.02)	2.19 (1.02)	2.34 (1.04)	2.33 (1.04)
	CDR sum of box	0.94 (1.52)	0.94 (1.52)	2.00 (2.72)	1.59 (2.43)
	Aβ-PET SUVR	0.874 (0.236)	0.874 (0.236)	0.941 (0.287)	0.914 (0.273)
	ΔAβ-PET SUVR	0.005 (0.065)	0.005 (0.065)	0.021 (0.108)	0.021 (0.107)
	Tau-PET SUVR	-	-	1.12 (0.262)	1.12 (0.215)
	Δ Tau-PET SUVR	-	-	-	0.029 (0.227)
	WMHs (log-transformed)	0.995 (0.335)	0.995 (0.335)	1.06 (0.37)	1.04 (0.387)
	Δ WMHs (log-transformed)	0.032 (0.30)	0.032 (0.30)	0.054 (0.381)	0.006 (0.271)

Note

Abbreviations: APOE ε4 = Apolipoprotein ε4, CN = Cognitively normal, MCI = Mild cognitive impairment, AD = Alzheimer’s disease, MMSE = Mini-mental state examination, CDR = Clinical dementia rating, SUVR = Standardized uptake value ratio, Aβ = Beta-amyloid, WMH = White matter hyperintensity

### Longitudinal relationship between cerebral Aβ deposition and WMH volume

**Fig. 2:**
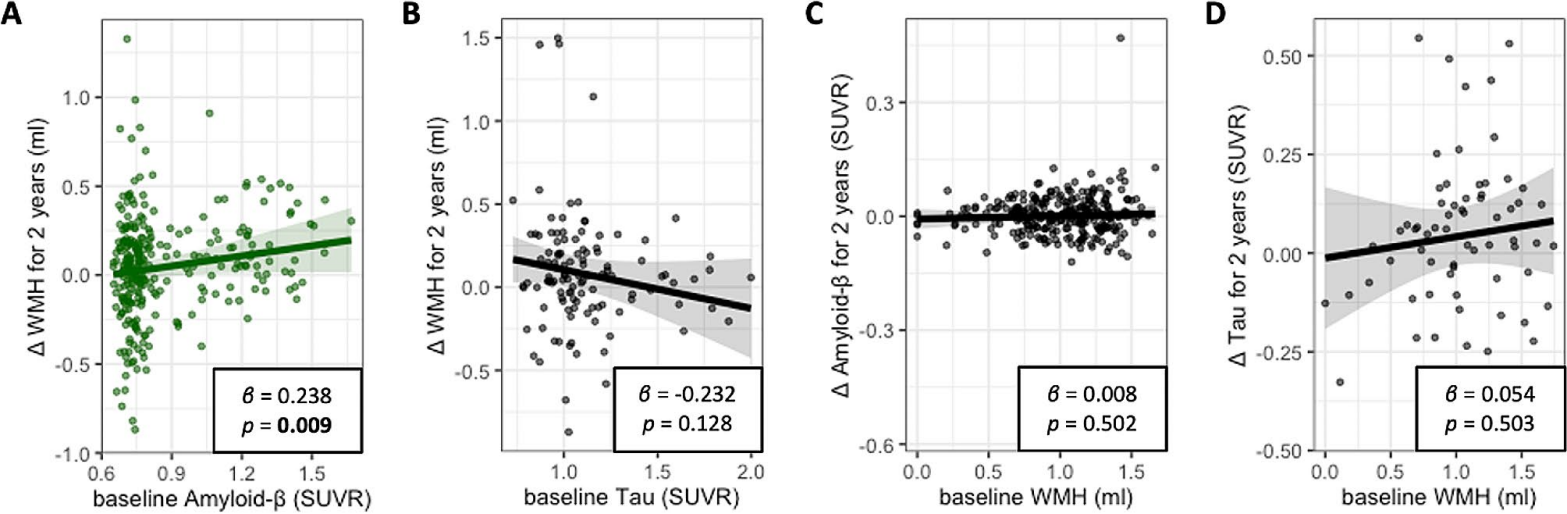
Bidirectional relation between Aβ or Tau deposition and WMH volume. **A**, Relation between Aβ and change of WMH for 2 years. Partial regression plot consisted of change of WMH for 2 years as dependent variable, and baseline Aβ as independent variable, adjusted for age, sex, APOE ε4 positivity, Vascular risk score, CDR sum of box and baseline WMH. **B**, Relation between tau and change of WMH for 2 years. Partial regression plot consisted of change of WMH for 2 years as dependent variable, and baseline tau as independent variable, adjusted for age, sex, APOE ε4 positivity, Vascular risk score, CDR sum of box and baseline WMH. **C**, Relation between WMH and change of Aβ for 2 years. Partial regression plot consisted of change of Aβ for 2 years as dependent variable, and baseline WMH as independent variable, adjusted for age, sex, APOE ε4 positivity, Vascular risk score, CDR sum of box and baseline Aβ. **D**, Relation between WMH and change of tau for 2 years. Partial regression plot consisted of change of tau for 2 years as dependent variable, and baseline WMH as independent variable, adjusted for age, sex, APOE ε4 positivity, Vascular risk score, CDR sum of box and baseline tau

Higher baseline cerebral Aβ deposition was associated greater increase of WMH volume for 2 years (*β* = 0.238, *p* = 0.009; Table 2; Fig. 2A). In contrast, there was no significant relationship between baseline WMH volume and change of Aβ deposition over 2 years (*β* = 0.008, *p* = 0.502; Table 2; Fig. 2C).

**Table 2:** Associations between Aβ or Tau deposition and WMH volume

Independent variable	Δ WMH volume ^a^		Δ Aβ ^b^		Δ Tau ^c^	
	β (95% CI)	*p*	β (95% CI)	*p*	β (95% CI)	*p*
baseline Aβ, SUVR	0.238 (0.059–0.418)	**0.009**	-	-	-	-
baseline Tau, SUVR	-0.232 (-0.532–0.068)	0.128	-	-	-	-
baseline WMH volume	-	-	0.008 (-0.016–0.032)	0.502	0.054 (-0.106–0.214)	0.503

Note

^a^ Adjusting for age, sex, APOE ε4 positivity, Vascular risk score, CDR sum of box, baseline WMH

^b^ Adjusting for age, sex, APOE ε4 positivity, Vascular risk score, CDR sum of box, baseline Aβ

^c^ Adjusting for age, sex, APOE ε4 positivity, Vascular risk score, CDR sum of box, baseline Tau

Abbreviations: Aβ = Beta-amyloid, SUVR = Standardized uptake value ratio, WMH = White matter hyperintensity

### Longitudinal relationship between cerebral tau deposition and WMH volume

Baseline tau deposition was not related with the 2-year change of WMH volume (*β* = -0.232, *p* = 0.128; Table 2; Fig. 2B). Baseline WMH volume was also not associated with change in tau deposition over 2 years (*β* = 0.054, *p* = 0.503; Table 2; Fig. 2D).

### Moderation on the relationship of cerebral Aβ deposition with WMH volume change

**Fig. 3:**
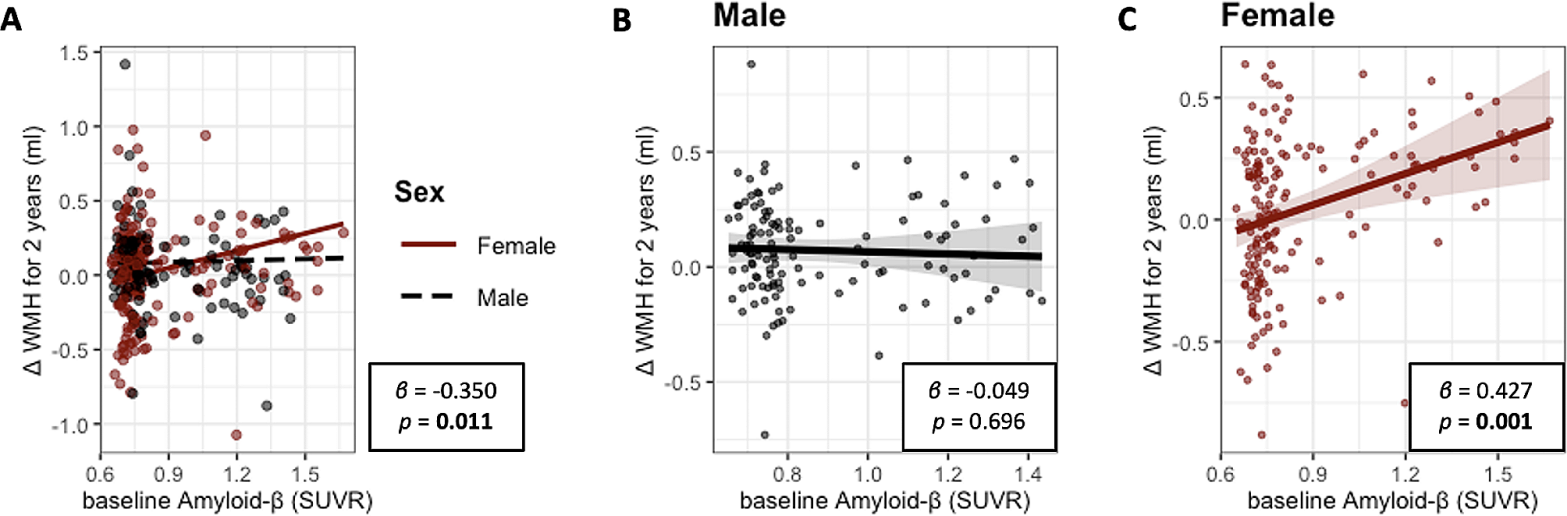
Moderating effect of sex on association between Aβ and WMH volume. **A**, Effect of sex on association between baseline Aβ and change of WMH volume for 2 years. Partial regression plot adjusted for age, APOE ε4 positivity, Vascular risk score, CDR sum of box and baseline WMH. Values in a box indicate coefficient and *p*-value of interaction term between baseline Aβ and sex. **B**, Relation between Aβ and change of WMH for 2 years in male. Partial regression plot consisted of change of WMH for 2 years as dependent variable, and baseline Aβ as independent variable, adjusted for age, APOE ε4 positivity, Vascular risk score, CDR sum of box and baseline WMH. **C**, Relation between Aβ and change of WMH for 2 years in female. Partial regression plot consisted of change of WMH for 2 years as dependent variable, and baseline Aβ as independent variable, adjusted for age, APOE ε4 positivity, Vascular risk score, CDR sum of box and baseline WMH.

We explored the moderating effects of age, sex, APOE ε4 positivity, VRS, CDR-SOB, or baseline WMH volume on the relationship between baseline Aβ deposition and change of WMH volume over 2 years which showed statistical significance. There was a significant baseline Aβ deposition × sex interaction effect on longitudinal change of WMH volume (*β* = -0.350, *p* = 0.011; Table 3; Fig. 3A). Subsequent subgroup analyses showed that higher baseline Aβ deposition was associated with greater increase of WMH volume over 2 years in female, but not in male (female: *β* = 0.427, *p* = 0.001; male: *β* = -0.049, *p* = 0.696; Fig. 3). In contrast, we did not find any significant interaction between Aβ and any other variables mentioned above (Table 3). While there was no significant association between baseline tau and WMH volume change, we further analyzed the moderating effects of age, sex, APOE ε4 positivity, VRS, CDR-SOB, or baseline WMH volume on the relationship between baseline tau deposition and change of WMH volume for comparison purposes. However, we did not find any significant moderating effects (Supplementary Table 2).

**Table 3:** Effect of moderator on association between baseline Aβ deposition and ΔWMH volume

	Δ WMH volume ^a^	
	β (95% CI)	*p*
Age × Baseline Aβ	-0.012 (-0.029–0.005)	0.173
Sex × Baseline Aβ	-0.350 (-0.621 – -0.079)	**0.011**
APOE ε4 positivity × Baseline Aβ	-0.058 (-0.369–0.253)	0.714
Vascular risk score × Baseline Aβ	-0.039 (-0.185–0.106)	0.595
CDR sum of box × Baseline Aβ	0.013 (-0.068–0.095)	0.748
Baseline WMH × Baseline Aβ	-0.083 (-0.566–0.400)	0.736

Note

^a^ Adjusting for age, sex, APOE ε4 positivity, Vascular risk score, CDR sum of box, baseline WMH

Abbreviations: APOE ε4 = Apolipoprotein ε4, CDR = Clinical dementia rating, SUVR = Standardized uptake value ratio, Aβ = Beta-amyloid, WMH = White matter hyperintensity

## Discussion

In the present study, we found that higher baseline cerebral Aβ deposition, but not tau deposition, was associated with greater increase of WMH volume over 2 years in older adults with diverse cognitive spectrum including normal cognition, MCI and AD dementia. In contrast, WMH volume at baseline did not show any relationship with the change of brain Aβ or tau deposition over 2 years.

Regarding the longitudinal relationship between Aβ and WMH volume, our findings suggest that baseline cerebral Aβ deposition may affect increase of WMH volume, whereas baseline WMH volume may not facilitate or precede the change of Aβ deposition. These observations were similar to a previous report which showed that lower baseline CSF Aβ_1−42_ was associated with increase of WMH volume over one year, while baseline WMH volume was not associated with change of CSF Aβ_1−42_ in individuals including CN, MCI and AD dementia [18]. In contrast, a study based on individuals with normal cognition reported that there was no relationship between baseline CSF Aβ_1−42_ and change of WMH volume [20]. Individuals with normal cognition, who possibly have a relatively low Aβ accumulation and small WMH volume change, might make it difficult to detect the relationship of Aβ with the increase of WMH volume. Additionally, in contrast to our result, another study which focused only on the influence of WMHs on Aβ change demonstrated that baseline WMH volume was significantly related with increase of cerebral Aβ deposition [19]. Although the study was conducted for cognitively unimpaired participants, they were followed up for relatively longer period (mean: 4.4 years; range from 1 to 8 years) compared to our study, which might have it possible to detect the relationship.

In regard of longitudinal tau-WMH relationship, we found no significant results for any direction of relationship, which is generally in line with previous reports based on cross-sectional analysis [13, 15]. In contrast, some diffusion tensor imaging (DTI) studies demonstrated a significant association between higher brain tau deposition and decreased white matter microstructural integrity as measured by fractional anisotropy (FA) or mean diffusivity (MD) [37–39]. Given these results, cerebral tau deposition may cause subtle or microstructural white matter damages that can be detected by FA or MD on DTI, but not by WMHs on T2 weighted MRI.

To date, the mechanism underlying the effect of Aβ on white matter injury (WMI) expressed as WMHs has not been clearly identified. Some evidence indicates that Aβ-induced death of oligodendrocytes and neuroinflammation may be primary mechanisms accounting for the effect of Aβ on white matter degeneration, demyelination in particular. Oligodendrocytes play an essential role in repairing impaired myelin sheath by remyelination. Cytotoxic effect of increasing Aβ can induce the death of oligodendrocytes, resulting in remyelination failure of damaged axons [40]. Increase of Aβ can also promote neuroinflammatory environment by fostering release of cytokine from astrocytes [41]. Neuroinflammation induced by Aβ can directly cause demyelination [42] and indirectly contribute to demyelination by interfering with an efficient remyelination of oligodendrocytes [43].

Additional exploratory analyses showed that the relationship between Aβ deposition and increase of WMH volume was significant only in female, but not in male. Such female predominant relationship may relate to drastic decrease in estrogen and progesterone during menopause and perimenopause [44]. The estrogen and progestogen can affect the production and activity of immune cells, and thus can be neuroprotective against AD and CVD pathogenesis [45, 46]. Weakened immune system resulted from decrease of these hormones can induce more vulnerable environment in which the toxicity of Aβ have a greater impact on brain in female. Some previous studies reported that effects of Aβ on neurodegeneration or cognitive decline were significant only in female, not male [47, 48]. A recent study also reported that WMH volume increases more rapidly in postmenopausal women compared to premenopausal women and men of the same age range [49].

The current study is unique in that it specifically investigated the directionality of the relationship between AD pathology and WMHs through longitudinal approaches. Nevertheless, it still had some limitations. First, sample size for the analysis of the relationship between cerebral tau deposition and WMH volume was relatively small, which might contribute to the non-significant results by reducing statistical power. Second, the 2-year follow-up period for longitudinal analysis also might not be sufficient to confirm some relationship between AD biomarkers and WMHs; for example, the relationship of baseline WMH volume with the change of brain Aβ or tau over 2 years. Further studies with a longer follow-up period are needed.

## Conclusions

The present findings in older adults with diverse cognitive spectrum suggested that baseline Aβ deposition contribute to the increase of WMHs over 2 years, especially in female, whereas WMIs appear not influence on longitudinal Aβ increase. The results also did not support any direction of influence between tau deposition and WMHs.

### Electronic supplementary material

Below is the link to the electronic supplementary material.

Supplementary Material 1

## Data Availability

The datasets used and analyzed during the current study are available from the corresponding author on reasonable request.

## Declarations

### Consortia

For the KBASE Research Group.

## Abbreviations

AD: Alzheimer’s disease
CVD: Cerebrovascular disease
WMH: White matter hyperintensity
WMI: White matter injury
Aβ: Beta-amyloid protein
p-tau: Phosphorylated tau protein
t-tau: Total tau protein
CSF: Cerebrospinal fluid
MRI: Magnetic resonance image
PET: Positron emission tomography
FLAIR: Fluid-attenuated inversion-recovery
DTI: Diffusion tensor imaging
FA: Fractional anisotropy
MD: Mean diffusivity
SUVR: Standardized uptake value ratio
ROI: Regions of interest
CN: Cognitively normal
MCI: Mild cognitive impairment
CDR: Clinical dementia rating
SOB: Sum of Boxes
VRS: Vascular risk factor scores
APOE ε4: Apolipoprotein ε4
KBASE: Korean Brain Aging Study for Early Diagnosis and Prediction of Alzheimer’s Disease
NIA-AA: National Institute on Aging and Alzheimer’s Association
DSM-IV-TR: Diagnostic and Statistical Manual of Mental Disorders 4th edition
CERAD-K: Korean version of the Consortium to Establish a Registry for Alzheimer’s Disease Assessment
