# Supplementary material for: Association between brain amyloid deposition and longitudinal changes of white matter hyperintensities
Source: Alzheimers Res Ther. 2024 Mar 7;16:50. doi: 10.1186/s13195-024-01417-8 (PMC10918927; doi:10.1186/s13195-024-01417-8)
Supplement: Supplementary file 1 — Supplementary Material 1 [file 13195_2024_1417_MOESM1_ESM.docx]

**Supplementary Table**

Supplementary Table 1. Comparison between male and female in characteristics

|  | Male (121) | Female (161) | T-test / chi-square | p value |
| --- | --- | --- | --- | --- |
| APOE ε4 carrier (+ / -) | 28 / 93 | 47 / 114 | 1.005 | 0.316 |
| Age, y | 71.5 (8.09) | 70.5 (7.75) | -1.06 | 0.289 |
| Education, y | 13.2 (4.51) | 9.56 (4.76) | -6.595 | **< .001** |
| MMSE score | 25.4 (3.77) | 23.9 (4.41) | -3.10 | **0.002** |
| Vascular risk score | 2.12 (1.03) | 2.25 (1.01) | 1.08 | 0.282 |
| CDR sum of box | 0.723 (1.26) | 1.10 (1.68) | 2.13 | **0.034** |
| Aβ-PET SUVR | 0.885 (0.227) | 0.865 (0.243) | -0.714 | 0.476 |
| WMH volume (ml) | 1.04 (0.318) | 0.965 (0.345) | -1.79 | 0.075 |
| △WMH volume (ml) | 0.051 (0.267) | 0.018 (0.323) | -0.931 | 0.353 |

*Note*.
Abbreviations: APOE ε4 = Apolipoprotein ε4, MMSE = Mini-mental state examination, CDR = Clinical dementia rating, Aβ = Beta-amyloid, SUVR = Standardized uptake value ratio, WMH = White matter hyperintensity.

Supplementary Table 2. Effect of moderator on association between baseline Tau deposition and △WMH volume

|  | △WMH volume ^a^ | |
| --- | --- | --- |
|  | β (95% CI) | p |
| Age × Baseline Tau | 0.007 (-0.027 – 0.041) | 0.681 |
| Sex × Baseline Tau | -0.082 (-0.651 – 0.488) | 0.777 |
| APOE ε4 positivity × Baseline Tau | 0.558 (-0.054 – 1.174) | 0.074 |
| Vascular risk score × Baseline Tau | 0.081 (-0.206 – 0.368) | 0.578 |
| CDR sum of box × Baseline Tau | -0.031 (-0.132 – 0.071) | 0.547 |
| Baseline WMH × Baseline Tau | -0.328 (-1.010 – 0.353) | 0.342 |

*Note*.

^a^ Adjusting for age, sex, APOE ε4 positivity, Vascular risk score, CDR sum of box, baseline WMH

Abbreviations: APOE ε4 = Apolipoprotein ε4, CDR = Clinical dementia rating, SUVR = Standardized uptake value ratio, WMH = White matter hyperintensity.

**Supplementary Figure**


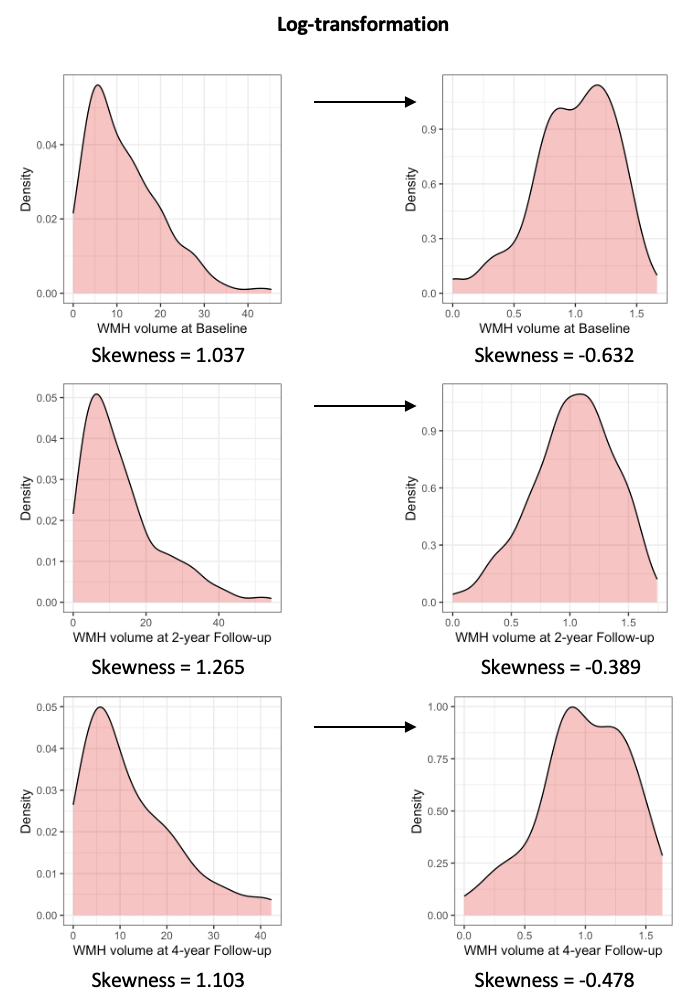


Supplementary Figure 1. The distribution of WMH volume and log-transformed WMH volume at baseline, 2-year, and 4-year Follow-up


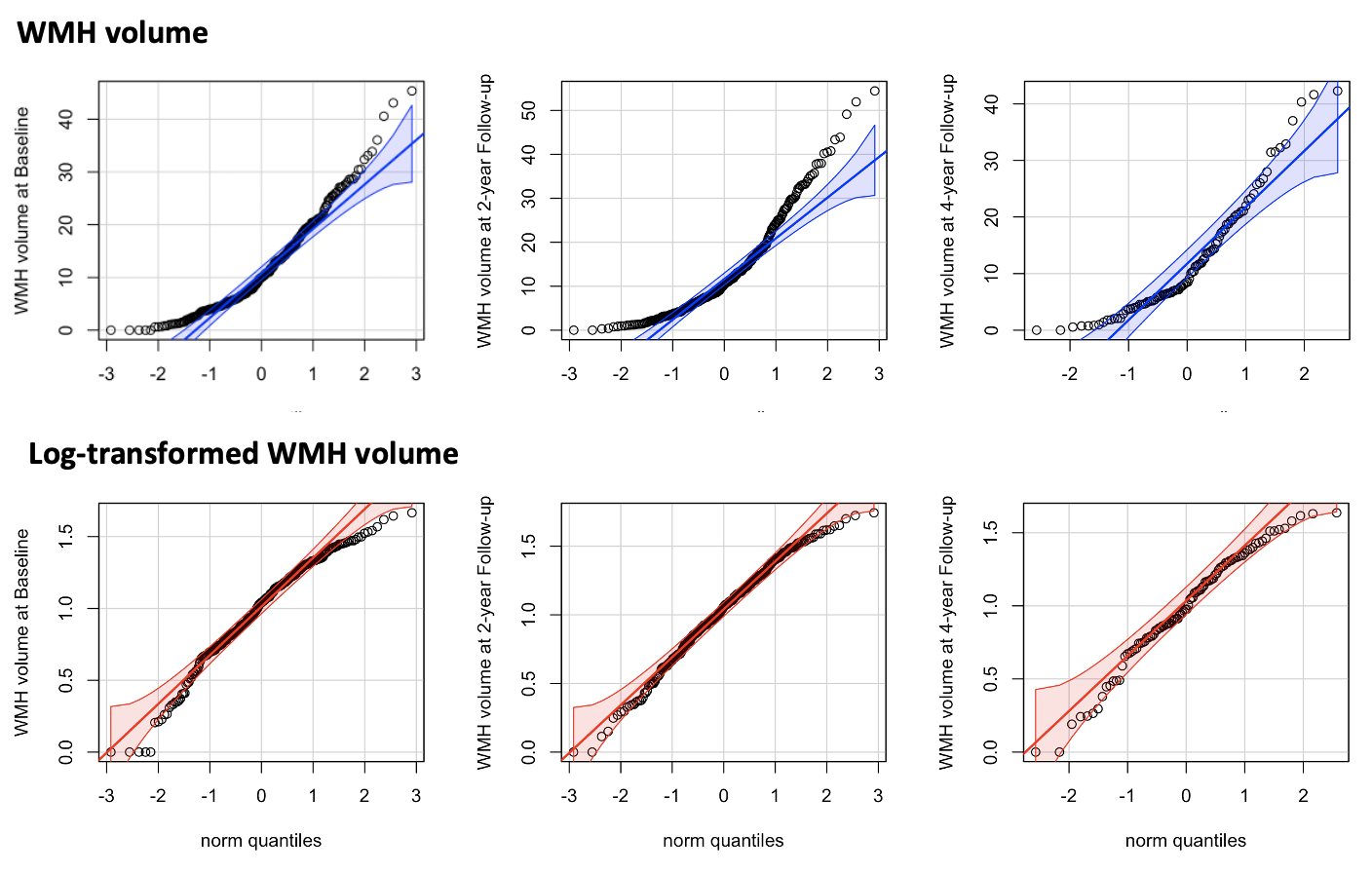


Supplementary Figure 2. Q-Q plots illustrating the distribution of WMH volume and log-transformed WMH volume at baseline, 2-year, and 4-year Follow-up

**Authors**

| Name | Location | Role | Contribution |
| --- | --- | --- | --- |
| Woo-Jin Cha, MA | Department of Neuropsychiatry, Seoul National University Hospital, Seoul, Republic of Korea | First Author | Designed and conceptualized the study; Acquired, analyzed and interpreted the data; Drafted and revised the manuscript for intellectual content |
| Dahyun Yi, PhD | Institute of Human Behavioral Medicine, Medical Research Center, Seoul National University, Seoul, Republic of Korea | Author | Acquired, analyzed and interpreted the data; Drafted and revised the manuscript for intellectual content |
| Hyejin Ahn, MA | Interdisciplinary program of cognitive science, Seoul National University College of Humanities, Seoul, Republic of Korea | Author | Acquired, analyzed and interpreted the data |
| Min Soo Byun, MD, PhD | Medical Research Center Seoul National University, Seoul, Republic of Korea | Author | Acquired, analyzed and interpreted the data |
| Yoon Young Chang, MD | Department of Psychiatry, Inje University Sanggye Paik Hospital, Seoul, Republic of Korea | Author | Acquired, analyzed and interpreted the data |
| Jung-Min Choi, MD | Department of Psychiatry, Inje University Sanggye Paik Hospital, Seoul, Republic of Korea | Author | Acquired, analyzed and interpreted the data |
| Kyungtae Kim, MD | Department of Psychiatry, Inje University Sanggye Paik Hospital, Seoul, Republic of Korea | Author | Acquired, analyzed and interpreted the data |
| Hyeji Choi, MD | Department of Psychiatry, Inje University Sanggye Paik Hospital, Seoul, Republic of Korea | Author | Acquired, analyzed and interpreted the data |
| Gijung Jung, RN, PhD | Seoul National University Hospital, Seoul, Republic of Korea | Author | Acquired, analyzed and interpreted the data |
| Koung Mi Kang | Department of Radiology, Seoul National University Hospital, Seoul, Republic of Korea | Author | Acquired, analyzed and interpreted the data |
| Chul-Ho Sohn, MD, PhD | Department of Radiology, Seoul National University Hospital, Seoul, Republic of Korea | Author | Acquired, analyzed and interpreted the data |
| Yun-Sang Lee, PhD | Department of Nuclear Medicine, Seoul National University College of Medicine, Seoul, Republic of Korea | Author | Acquired, analyzed and interpreted the data |
| Yu Kyeong Kim, MD, PhD | Department of Nuclear Medicine, Seoul Metropolitan Government‑Seoul National University Boramae Medical Center, Seoul, Republic of Korea | Author | Acquired, analyzed and interpreted the data |
| Dong Young Lee, MD, PhD | Department of Neuropsychiatry, Seoul National University Hospital, Seoul, Republic of Korea | Corresponding Author | Designed and conceptualized the study; Acquired, analyzed and interpreted the data; Drafted and revised the manuscript for intellectual content |

**Coinvestigators**

| Name | Location | Role | Contribution |
| --- | --- | --- | --- |
| Dong Young Lee, MD, PhD | Seoul National University College of Medicine | Principal Investigator | Designed and conceptualized the cohort study; Led and supervised the cohort study; coordinated communication among study cores and study sites; acquired funding |
| Min Soo Byun, MD, PhD | Medical Research Center Seoul National University | Core PI Clinical & Executive | Supervised and coordinated the Clinical and Executive core of the cohort study |
| Dahyun Yi, PhD | Medical Research Center Seoul National University | Core PI Neuropsychology | Supervised and coordinated the Neuropsychological Core of the cohort study |
| Yu Kyeong Kim, MD, PhD | SMG-SNU Boramae Medical Center | Core PI PET | Supervised and coordinated the PET Core of the cohort study |
| Chul-Ho Sohn, MD, PhD | Seoul National University College of Medicine | Core PI MRI | Supervised and coordinated the MRI Core of the study |
| Inhee Mook-Jung, PhD | Seoul National University College of Medicine | Core PI Biomarker | Supervised and coordinated the Biomarker Core of the study |
| Murim Choi, PhD | Seoul National University | Core PI Genetics | Supervised and coordinated the Genetic Core of the study |
| Yu Jin Lee, MD, PhD | Seoul National University College of Medicine | Core PI Sleep | Supervised and coordinated the Sleep Core of the study |
| Seokyung Hahn, PhD | Seoul National University College of Medicine | Core PI Biostatistics | Supervised and coordinated the Biostatistics Core of the study |
| Hyun Jung Kim, MD | Changsan Convalescent Hospital | co-investigator | Performed clinical assessment of participants and quality control of the clinical data |
| Mun Young Chang, MD, PhD | Chung-Ang University College of Medicine | co-investigator | Coordinated an add-on study of the main cohort study |
| Seung Hoon Lee, MD, PhD | Myongji Hospital | co-investigator | Performed clinical assessment of participants and quality control of the clinical data |
| Na Young Han, MD | Dongrae Medical Center | co-investigator | Performed clinical assessment of participants and quality control of the clinical data |
| Jisoo Pae, MD, PhD | Genome & Company | co-investigator | Coordinated an add-on study of the main cohort study |
| Hansoo Park, MD, PhD | Genome & Company | co-investigator | Coordinated an add-on study of the main cohort study |
| Jee Wook Kim, MD, PhD | Hallym University Dongtan Sacred Heart Hospital | co-investigator | Coordinated a study site and performed participants recruitment and quality control of the clinical data |
| Jong-Min Lee, PhD | Hanyang University | co-investigator | Coordinated an add-on study of the main cohort study |
| Dong Woo Lee, MD, PhD | Inje University Snaggye Paik Hospital | co-investigator | Coordinated a study site and recruited participants of the cohort study |
| Bo Kyung Sohn, MD, PhD | Inje University Snaggye Paik Hospital | co-investigator | Coordinated a study site and recruited participants of the cohort study, performed clinical data analysis |
| Seok Woo Moon, MD, PhD | Konkuk University Chungju Hospital | co-investigator | Coordinated a study site and performed clinical data analysis |
| Hyewon Baek, MD | Kyunggi Provincial Hospital for the Elderly | co-investigator | Performed clinical assessment of participants and quality control of the clinical data |
| Yoon-Keun Kim, MD, PhD | MD Healthcare Inc. | co-investigator | Coordinated an add-on study of the main cohort study |
| Jong-Won Kim, MD, PhD | Samsung Medical Center | co-investigator | Supervised and performed genetic analysis |
| Seung-Ho Ryu, MD, PhD | Konkuk University Medical Center | co-investigator | Coordinated a study site and recruited participants |
| Shin Gyeom Kim, MD, PhD | Soonchunhyang University Hospital Bucheon | co-investigator | Coordinated a study site and performed clinical data analysis |
| Jong Inn Woo, MD, PhD | Seoul National University | co-investigator | Supervised and advised the cohort study |
| Sang Eun Kim, MD, PhD | Seoul National University Bundang Hospital | co-investigator | Coordinated the production of PET radiotracer |
| Gi Jeong Cheon, MD, PhD | Seoul National University Hospital | co-investigator | Coordinated the acquisition of the PET data |
| Koung Mi Kang, MD, PhD | Seoul National University Hospital | co-investigator | Participated in the acquisition and clinical interpretation of the MRI/MRA data |
| Jee-Eun Park, MD, PhD | Seoul National University Hospital | co-investigator | Performed clinical and sleep-related data analysis |
| Hyeong Gon Yu, MD, PhD | Seoul National University Hospital | co-investigator | Coordinated an add-on study of the main cohort study |
| Jun-Young Lee, MD, PhD | SMG-SNU Boramae Medical Center | co-investigator | Coordinated a study site and performed participants recruitment |
| Hyo Jung Choi, MD | SMG-SNU Boramae Medical Center | co-investigator | Performed clinical assessment of participants and quality control of the clinical data |
| Young Min Choe, MD, PhD | Hallym University Dongtan Sacred Heart Hospital | co-investigator | Performed recruitment and clinical assessment of participants and monitoring of the clinical data |
| Kwangsoo Kim, Ph.D | Seoul National University Hospital | co-investigator | Supervised and performed biostatistics data analysis |
| So Yeon Jeon, MD, PhD | Chungnam National University Hospital | co-investigator | Coordinated participant recruitment and follow-up, performed clinical assessment of participants, quality control of the clinical data |
| Woo Jin Kim, MD, PhD | Seoul National University Hospital | research fellow | Performed clinical assessment of participants and quality control of the clinical data |
| Kang Ko, MD | National Health Insurance Service Ilsan Hospital | co-investigator | Performed clinical assessment of participants and quality control of the clinical data |
| Jun Ho Lee, MD, PhD | Seoul National University Hospital | research fellow | Coordinated participant recruitment and follow-up, performed clinical assessment of participants, quality control of the clinical data analysis |
| Sung Wook Park, MD, PhD | Seoul National University Hospital | research fellow | Performed an add-on study and data analysis |
| Gijung Jung, RN, PhD | Seoul National University | research coordinator | Coordinated participants recruitment, follow-up and assessment among sites, performed clinical assessment of participants and data monitoring |
| Haejung Joung | Seoul National University Hospital | psychologist | Performed neuropsychological assessment of participants. quality control and preprocessing of the data |
| HyeJin Ahn | Seoul National University Hospital | psychologist | Performed neuropsychological assessment of participants. quality control and preprocessing of the data |
| Han Na Lee | Seoul National University Hospital | research coordinator | Coordinated participants recruitment, follow-up and assessment among sites, performed clinical assessment of participants and data monitoring |
| Joon Hyung Jung, MD | Chungbuk National University Hospital | co-investigator | Performed clinical assessment of participants and quality control of the clinical data |
| Gihwan Byeon, MD | Kangwon National University Hospital | co-investigator | Performed clinical assessment of participants and quality control of the clinical data |
| Kiyoung Sung, MD | Seoul National University Hospital | research fellow | Performed the clinical data analysis |
| Dong Kyun Han, MD | Seoul National University Hospital | research fellow | Performed clinical assessment of participants and quality control of the clinical data |
| Seung Min Han, MD | Seoul National University Hospital | research fellow | Performed clinical assessment of participants and quality control of the clinical data |
| Min Jung Kim, MD | Seoul National University Hospital | research fellow | Performed clinical assessment of participants and quality control of the clinical data |
| Min Jae Kim, MD | Soonchunhyang University Hospital | co-investigator | Performed clinical assessment of participants and quality control of the clinical data |
| Nayeong Kong, MD, PhD | Keimyung University Dongsan Hospital | co-investigator | Performed clinical assessment of participants and quality control of the clinical data |
| Seo Hee Park, MD | Seoul National University Hospital | research fellow | Performed clinical assessment of participants and quality control of the clinical data |
| Mimi Kim, RN | Seoul National University Hospital | research coordinator | Coordinated participants recruitment, Performed clinical assessment of participants |
| Woo-Jin Cha, MA | Seoul National University Hospital | psychologist | Performed neuropsychological assessment of participants. quality control and preprocessing of the data |
| Hyeryeon Yeom, MA | Seoul National University Hospital | psychologist | Performed neuropsychological assessment of participants. quality control and preprocessing of the data |
| Yoon Young Chang, MD | Inje University Sanggye Baek Hospital | co-investigator | Performed clinical assessment of participants and quality control of the clinical data |
| Musung Keum, MD, PhD | Seoul National University Hospital | research fellow | Performed clinical assessment of participants and quality control of the clinical data |
| Min Jeong Kim, RN | Seoul National University Hospital | research coordinator | Coordinated participants recruitment, Performed clinical assessment of participants |
| Donghee Kim, RN | Seoul National University Hospital | research coordinator | Coordinated participants recruitment, Performed clinical assessment of participants |
| Kyungtae Kim, MD | Seoul National University Hospital | research fellow | Performed clinical assessment of participants and quality control of the clinical data |
| Jeongmin Choi, MD | Seoul National University Hospital | research fellow | Performed clinical assessment of participants and quality control of the clinical data |
| Hye Ji Choi, MD | Seoul National University Hospital | research fellow | Performed clinical assessment of participants and quality control of the clinical data |
| Bae han sol, RN | Seoul National University Hospital | research coordinator | Coordinated participants recruitment, Performed clinical assessment of participants |
| Dohyun Woo, MA | Seoul National University Hospital | psychologist | Performed neuropsychological assessment of participants. quality control and preprocessing of the data |
| Seunghyuk Ha, MA | Seoul National University Hospital | psychologist | Performed neuropsychological assessment of participants. quality control and preprocessing of the data |
| Eun Suk Song, RN | Seoul National University Hospital | research coordinator | Coordinated participants recruitment, Performed clinical assessment of participants |
